# The α-subunit of the rice heterotrimeric G protein, RGA1, regulates drought tolerance during the vegetative phase in the dwarf rice mutant *d1*

**DOI:** 10.1093/jxb/erw183

**Published:** 2016-05-18

**Authors:** Ángel Ferrero-Serrano, Sarah M. Assmann

**Affiliations:** Biology Department, Penn State University, 208 Mueller Laboratory, University Park, PA 16802, USA

**Keywords:** *d1*, drought, heterotrimeric G protein, leaf temperature, *Oryza sativa*, RGA1, rice.

## Abstract

Essential in the Green Revolution was the development of high-yielding dwarf varieties of rice (*Oryza sativa* L.), but their selection was not based on responses to water limitation. We studied physiological responses to progressive drought of the dwarf rice mutant, *d1*, in which the *RGA1* gene, which encodes the GTP-binding α-subunit of the heterotrimeric G protein, is non-functional. Wild-type (WT) plants cease net carbon fixation 11 days after water is withheld, while *d1* plants maintain net photosynthesis for an additional week. During drought, *d1* plants exhibit greater stomatal conductance than the WT, but both genotypes exhibit the same transpirational water loss per unit leaf area. This is explained by a smaller driving force for water loss in *d1* owing to its lower leaf temperatures, consistent with its more erect architecture. As drought becomes more severe, WT plants show an accelerated decline in photosynthesis, which may be exacerbated by the higher leaf temperatures in the WT. We thus show how a rice mutant with dwarf and erect leaves has a decreased susceptibility to water stress. Accordingly, it may be useful to incorporate *RGA1* mutation in breeding or biotechnological strategies for development of drought-resistant rice.

## Highlight

The rice *d1* Gα mutant with dwarf and erect leaves exhibits a lower leaf temperature and reduced susceptibility to water stress.

## Introduction

Rice (*Oryza sativa* L.) is the staple food for more than half of the world’s population and is one of the three major cereal crops. In 2012, the total harvested area for rice was >150 Mha and its annual production was >700 Mt, of which 55% of the area and 45% of the production corresponded to low-income food deficit countries (LIFDCs), which are those lacking the resources not only to import but also to produce sufficient amounts of food domestically (FAOstat, 2012). The importance of rice to global agriculture was recognized during the Green Revolution (Evenson and Gollin. 2003), when grain production increased dramatically as a result of the introduction of new high-yielding varieties of rice and wheat for use in the developing world. Dwarf rice varieties with reduced stature and other associated traits such as erect leaves were a cornerstone of Green Revolution breeding programs (Duvick and Cassman, 1999; Khush, 2001). These dwarf varieties provide the advantages of an increased harvest index and decreased lodging (Hedden 2003).

Dwarf forms of japonica rice were reported as early as the first half of the 19th century by Japanese naturalist Iwasaki Tsunemasa (Iwasaki, 1786–1842). Traditionally, two main groups of dwarf japonica rice have been described: ‘Daikoku’, which is the most common and is named after Daikokuten, the Japanese deity of agriculture and rice, and ‘Bonsai’ (Nagai, 1959). *d1* was among the first rice mutants to be identified by segregation analysis (Akemine, 1925). It was originally found in Hokkaido in 1912 as a natural mutant originating from the Akage cultivar (Nagai, 1959), an inbred line developed from a landrace in order to increase grain size (Shomura *et al.*, 2008). Later on, further segregation analysis suggested the division of what until then was known as the ‘Daikoku’ type into different linkage groups, and *d1* was classified within the ‘Daikoku’ dwarf subgroup (Nagao, 1952). *d1* plants exhibit short, broad erect leaves, erect and compact panicles, and small rounded seeds. More recently, the *d1* phenotype has been identified as resulting from mutation in the gene encoding RGA1, the α-subunit of the heterotrimeric G protein (Ashikari *et al.*, 1999; Fuijisawa et al., 1999); its initial identification in 1925 possibly makes *d1* the first G protein mutant organism ever recorded, as well as one for which agronomic relevance was evident even before it was known to be a G protein mutant. Multiple *RGA1* mutants have now been identified, and all of them exhibit a similar phenotype (Oki *et al.*, 2009a). Rice *RGA1* encodes a 380 amino acid protein and, in this study, we investigated the *d1-1* mutant in cultivar Taichung 65 (T65) in which there is a 2bp deletion in *RGA1* that results in a protein null phenotype based on immunoblot analysis (Oki *et al.*, 2009b).

Heterotrimeric G proteins function as molecular switches that transduce signals from receptor molecules to effector molecules. The inactive G protein is a trimer consisting of a Gα subunit with bound GDP, and a Gβγ dimer. The activated, GTP-bound Gα dissociates from Gβγ, facilitating interaction of Gα and Gβγ with downstream effectors until the GTPase activity of Gα hydrolyzes GTP, leading to trimer reassembly (Assmann, 2002; Jones and Assmann, 2004; Perfus-Barbeoch *et al.*, 2004; Urano *et al.*, 2013). In contrast to mammalian genomes, which encode multiple genes for each G protein subunit, plant genomes contain fewer G protein genes. The rice genome contains a single canonical Gα gene, *RGA1* (Ishikawa *et al.*, 1995); a single Gβ gene, *RGB1* (Ishikawa *et al.* 1996); four Gγ subunit genes, *RGG1*, *RGG2*, *GS3*, and *DEP1* (Kato *et al.*, 2004; Fan *et al.*, 2006; Huang *et al.*, 2009); and a fifth related Gγ gene or pseudogene *OsGGC2* (Botella, 2012). Recently, the three extra-large G proteins (XLGs) of *Arabidopsis thaliana* L. (Heyn), were also found to couple with Gβγ (Chakravorty *et al.*, 2015; Maruta *et al.*, 2015); rice has three candidate *XLG* genes (*XLG1*, *XLG2*, and *XLG3*; Maruta *et al.*, 2015).

Dwarf varieties that constituted the paradigm of the Green Revolution were later described as those with alterations in gibberellin (GA) response modulators (Peng *et al.*, 1999; Sakamoto *et al.*, 2003). More recently, a complementary strategy for improving yield in rice has also been proposed, consisting of targeting the brassinosteroid pathway, which influences both plant height and leaf angle (Sakamoto *et al.*, 2005); it has been suggested that erect leaves are also a trait of interest in post-Green Revolution varieties (Sinclair and Sheehy, 1999). RGA1 is involved in both GA and brassinosteroid responses. Loss-of-function mutation of the *RGA1* gene impairs GA induction of both α-amylase in the aleurone layer and elongation of certain internodes (Ueguchi-Tanaka *et al.*, 2000). Additionally, *RGA1* has been found to be epistatic to *SLR*, encoding a DELLA protein acting as a repressor of GA signaling (Ueguchi-Tanaka *et al.*, 2000). However, *d1* plants do not completely lose GA sensitivity, suggesting the existence of a parallel G protein-independent GA pathway (Ueguchi-Tanaka *et al.*, 2000). RGA1 may also be involved in brassinosteroid signaling (Yang *et al.*, 2003; Wang *et al.*, 2006). Although the role that RGA1 plays in BRI1-mediated brassinosteroid signaling is under debate (Oki *et al.*, 2009b; Hu *et al.*, 2013), *d1* mutants show reduced lamina bending in response to brassinosteroid application (Oki *et al.*, 2009b), and mutants in *Taihu Dwarf1* (*TUD1*), a gene encoding a U-box E3 ubiquitin ligase that physically interacts with RGA1, also show reduced brassinosteroid sensitivity (Hu *et al.*, 2013).

Despite the attention that *d1* mutants have received, they have not been the focus of breeding studies and no detailed physiological studies have been performed on this mutant, or on its responses to environmental cues. In *A. thaliana*, the sole Gβ subunit, AGB1, regulates yield stability in response to water availability (Nilson and Assmann, 2010a), and the sole canonical Gα subunit, GPA1, is a regulator of transpiration efficiency (Nilson and Assmann, 2010b), but the relationship between heterotrimeric G proteins and water relations in rice has not been explored. Accordingly, the main objective of the present study was to characterize the responses of *d1* plants to water limitation. Since even a few days of drought can impair seedling establishment in the field (O’Toole *et al.*, 1978), we sought to assess responses of young *d1* mutant plants to progressive drought. Despite the fact that drought is a major constraint on yield, and the single greatest cause of yield loss in rainfed rice, breeding programs to date have focused on increasing rice yields in irrigated conditions (Tuong and Bouman, 2003). The results reported here suggest that the *d1* mutation should be considered in future breeding programs aiming to ameliorate yield loss under non-irrigated conditions.

## Materials and methods

### Plant growth conditions

Wild-type (WT) and *d1* mutant plants were grown in a greenhouse in 6 inch pots containing Metro-mix 360 potting mixture. Greenhouse temperatures averaged 30 °C during the day and 20 °C during the night in a 16:8 day:night cycle with light supplied as natural daylight supplemented with 1000W metal halide lamps (Philips Lighting Co., Somerset, NJ, USA) for the duration of the light cycle. Light intensity was ~500 μmol m^−2^ s^−1^ photosynthetic photon flux density (PPFD).

### Treatments

**Fig. 1.**
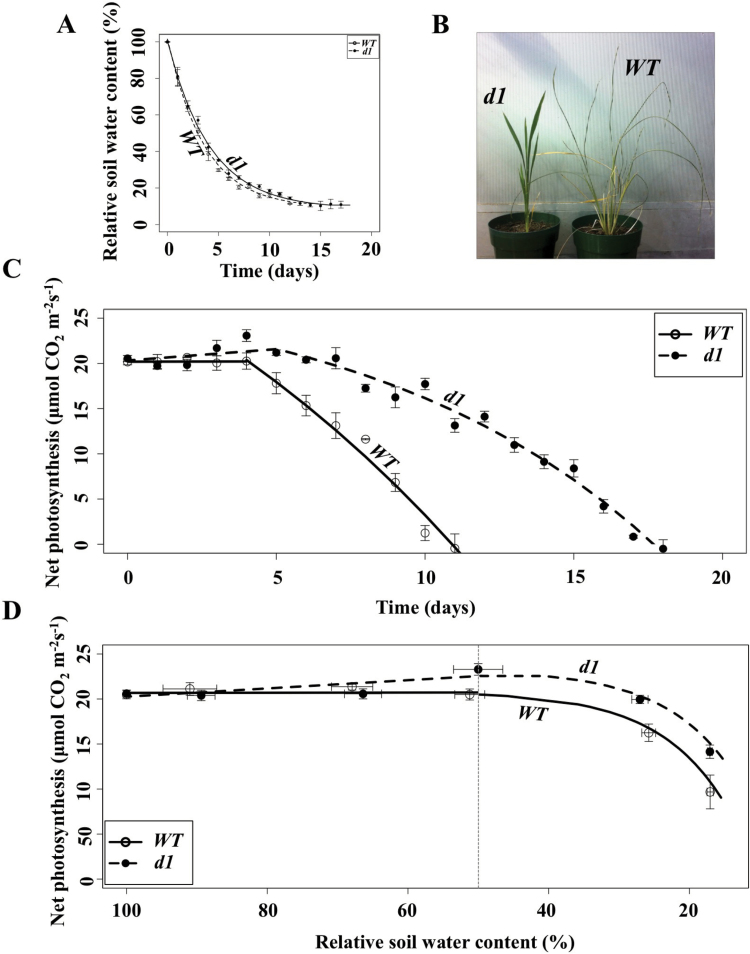
Wild-type (WT) plants are notably more affected by water limitation than *d1* plants. (A) Pots in which WT plants were grown lost water from the soil at a rate identical to that for pots in which *d1* plants were grown. Fits were obtained using a decay function (Supplementary Table S1). Open circles are data points for the WT, and filled circles are data points for *d1*. Model fits are shown with a solid line for the WT and a dashed line for *d1*. This notation is used in all of the figures. (B) Photograph of representative WT and *d1* plants taken 14 d after water was withheld. (C) WT plants exhibit a steeper decline in CO_2_ fixation as soil dries out. Fits were made using a linear fit for the response until day 4 (Supplementary Table S2) and a saturating exponential fit for the response from day 4 onward (Supplementary Table S3). (D) Net photosynthesis increases in *d1* as relative soil water content (RSWC) declines to ~50% while net photosynthesis for the WT remains constant over this period. Subsequently, net photosynthesis declines more gradually in *d1* than in the WT. Data were fitted to a linear model to characterize the results from 100% RSWC to 50% RSWC (Supplementary Table S4), and to a saturating exponential equation for the response from 50% to 15% RSWC (Supplementary Table S5). The vertical dotted line indicates 50% RSWC. (This figure is available in colour at *JXB* online.)

There were 40 replicates per genotype, and then the entire experiment was replicated, for a total of 80 plants per genotype. Plants were maintained for the first 60 d after emergence with soil well watered so that the relative soil water content (RSWC) never dropped below 75%. Day 60 was designated as day 0 of the drought treatment; plants were watered to soil water field capacity for the last time and then the soil was left to dry progressively (Fig. 1A). Individual plants were randomly selected for non-destructive physiological measurements throughout the desiccation period, and the individual sample size for each measured variable is detailed in the respective Supplementary tables. Each physiological measurement was accompanied by a measurement of the RSWC, taken immediately before the physiological measurement using a Campbell Scientific TDR 100 system (Campbell Scientific Inc., Logan, UT, USA) with a custom-made probe of 10cm length.

### Physiological measurements

For gas exchange measurements, regions of flag leaves at the point of maximal width were placed inside the chamber of a portable gas exchange system (LI-COR 6400 IRGA with an integrated 6400-40 2cm^2^ leaf chamber fluorometer, Li-COR, Inc., Lincoln, NE, USA). The leaf chamber temperature was kept constant at 30 °C for all gas exchange measurements. Airflow in the chamber was adjusted to 300 μmol s^−1^. Throughout the experiment, steady-state measurements of the different gas exchange parameters were performed after flag leaves were allowed to acclimate to an air CO_2_ partial pressure (*C*_a_) of 400 μl l^−1^ and 500 μmol m^−2^ s^−1^ PPFD as provided by the Li-COR 6400-40 red/blue LED system, with blue light accounting for 10% of the total photon flux.

Light response curves (*A*/*I*) were obtained by determining photosynthetic rates in flag leaves over a range of irradiances sequentially stepped down from high to low (2000, 1500, 1000, 750, 500, 300, 200, 100, and 0 μmol m^−2^ s^−1^ PPFD). Dual Peltier devices on the sensor head prevented leaf heating at higher irradiances, keeping the block temperature constant at 30 °C. Light-saturated photosynthetic capacity (*A*_sat_) and dark respiration (*R*_d_) were calculated by fitting the data to the model of Hanson *et al.* (1987). Apparent quantum yield (Φ) was estimated as the first derivative of the fitted equation at PPFD=0 (Hanson *et al.*, 1987).

To measure photosynthesis as a function of intercellular CO_2_ concentration (*C*_i_) in *A*/*C*_i_ curves, flag leaves were allowed to acclimate to low *C*_a_ (100 μl l^−1^ CO_2_) in the leaf chamber before gradually increasing *C*_a_. Gas exchange rates were determined across a series of *C*_a_: 100, 150, 250, 350, 400, 500, 700, and 900 μl l^−1^ CO_2_. These measurements were conducted after acclimation to saturating light conditions (1500 μmol m^−2^ s^−1^ PPFD as determined from the light response curves); this light intensity was chosen because both genotypes reached light saturation of photosynthesis at this value. We define light- and CO_2_-saturated photosynthetic capacity derived from these data as *A*_max_.

Leaf temperatures of WT and *d1* plants were determined at a point in the middle of the flag leaf of the primary tiller from thermal images obtained using a FLIR T620 thermal imaging camera (FLIR Systems, USA). The system incorporates an uncooled microbolometer with a spectral range of 7.8–14 μm, 640×480 pixels resolution with a thermal sensitivity <50 mK in the range of temperatures over which these images were obtained. Emissivity was set at 0.95. The set-up consisted of a black sheet in the background with plants set at a 250cm distance from the camera. Infrared thermal images were taken between 12:00h and 13:00h. Light intensity was ~500 μmol m^−2^ s^−1^ PPFD and air temperature was 30 °C. Images were analyzed for leaf temperature using the Flir Quick Report software with suitable scale and palette ranges.

Total leaf area per plant was determined by ImageJ (http://rsb.info.nih.gov/ij/) analysis of digital images obtained from flattened leaves. Once physiological measurements were obtained, plants were immediately harvested, roots were washed, and above- and below-ground biomass was measured separately after drying the plant material at 60 °C for 48h.

### Data analysis

The nlme package of Rgui (R Development Core Team, 2015) was used for statistical analysis and two-dimensional model fitting. Fittings were based on the most adequate model after inspection of residuals, Akaike information criterion (AIC), Bayesian information criterion (BIC), and examination of systematic lack of fit. The selected model in every instance was chosen based on these criteria and on simplicity in order to facilitate the discussion of meaningful parameters. Model selection also aimed to maintain consistency in the representation of different parameters exhibiting the same trend (i.e. saturating exponential, decay, or linear relationships) for different parameters. Three-dimensional fits of the relationships between *A*/*I* and *A*/*C*_i_ curves and soil relative water content were obtained using Tablecurve3D 4.0 (Systat Software, Inc.). Details on the equations fitted for each analysis, sample size, goodness of fit, and the estimated parameters can be found in Supplementary Data S1 at *JXB* online.

In order to normalize the data and focus attention on water availability rather than chronology, we focus on the differences between the WT and *d1* as a function of RSWC. All differences discussed are statistically significant (see Supplementary Data S1), unless explicitly stated otherwise.

**Fig. 2.**
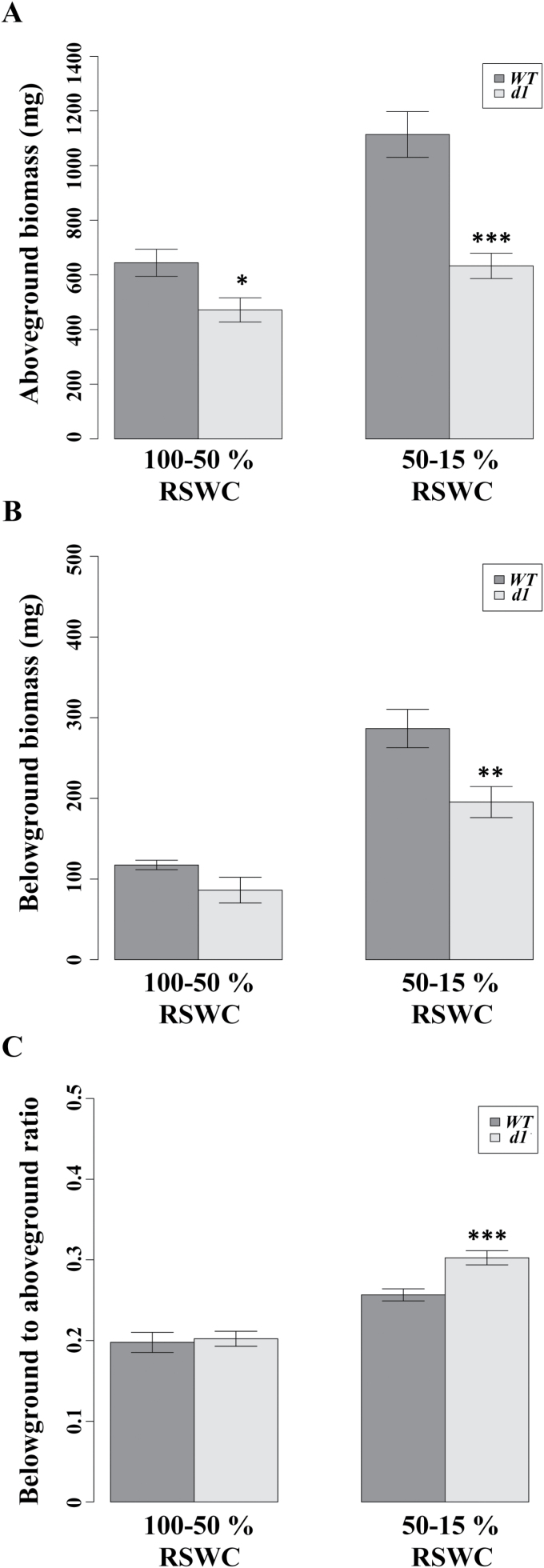
Wild-type (WT) plants in the absence of drought exhibit a larger biomass than *d1*; with drought, the root to shoot ratio is increased in *d1* to a greater extent than in the WT. (A) WT plants exhibited greater above-ground and (B) below-ground biomass consistent with the dwarf phenotype of *d1*. (C) In the absence of drought, both genotypes exhibit similar ratios of below-ground to above-ground biomass. As water becomes limiting, the root to shoot ratio increases for both genotypes, but to a greater extent in *d1.* Bars represent means ±SE.

**Fig. 3.**
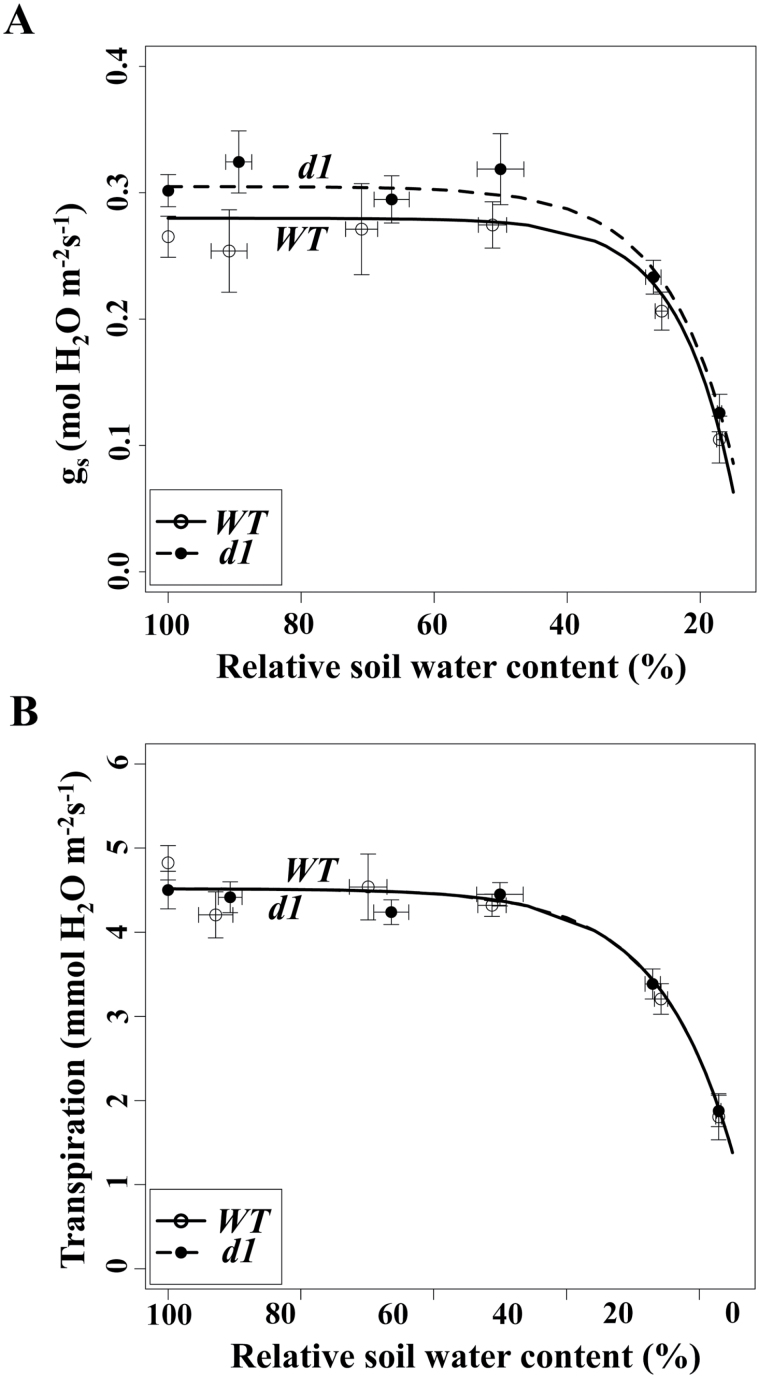
The wild type (WT) exhibits a lower stomatal conductance, but greater transpirational water loss for any given conductance than *d1*. (A) The WT exhibits a lower stomatal conductance than *d1* under well-watered conditions; conductances converge as the relative soil water content (RSWC) decreases. Data were fitted to a saturating exponential model (Supplementary Table S6). (B) Transpiration rates did not differ between the WT and *d1*. Data were fitted to a saturating exponential equation (Supplementary Table S7).

**Fig. 4.**
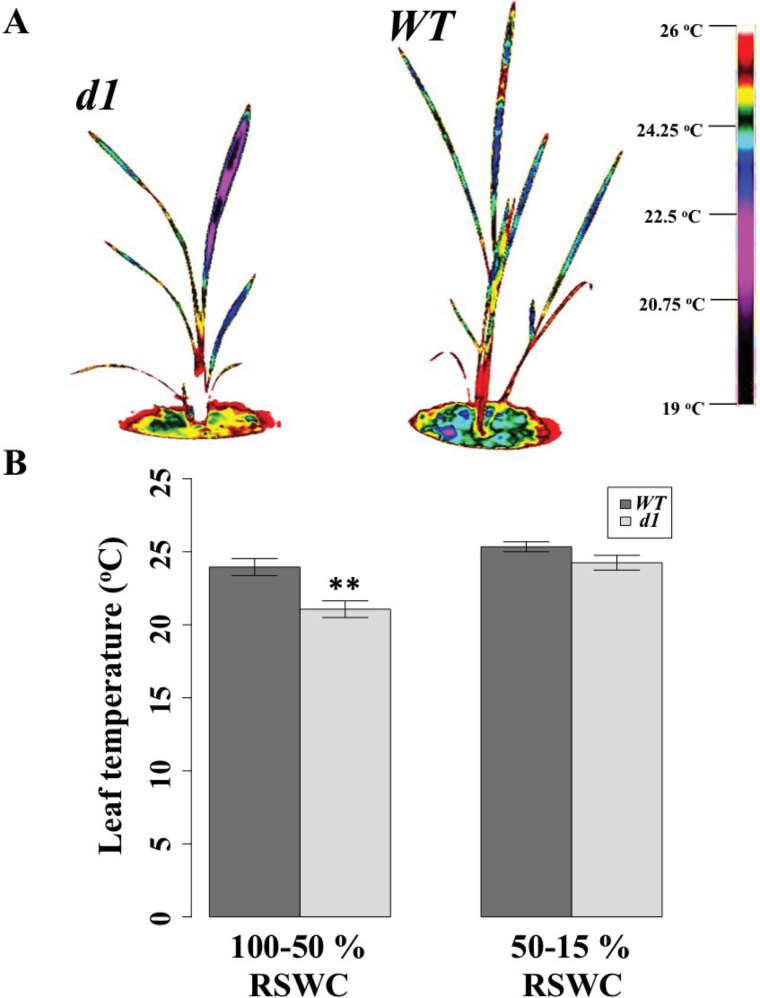
Wild-type (WT) plants exhibit higher leaf temperatures. (A) Thermal images of WT and *d1* plants under well-watered conditions. The scale on the right denotes temperatures shown in the images. (B) Leaf temperatures increase in both genotypes as relative soil water content (RSWC) declines from 30% but are always higher in the WT. (This figure is available in colour at *JXB* online.)

**Fig. 5.**
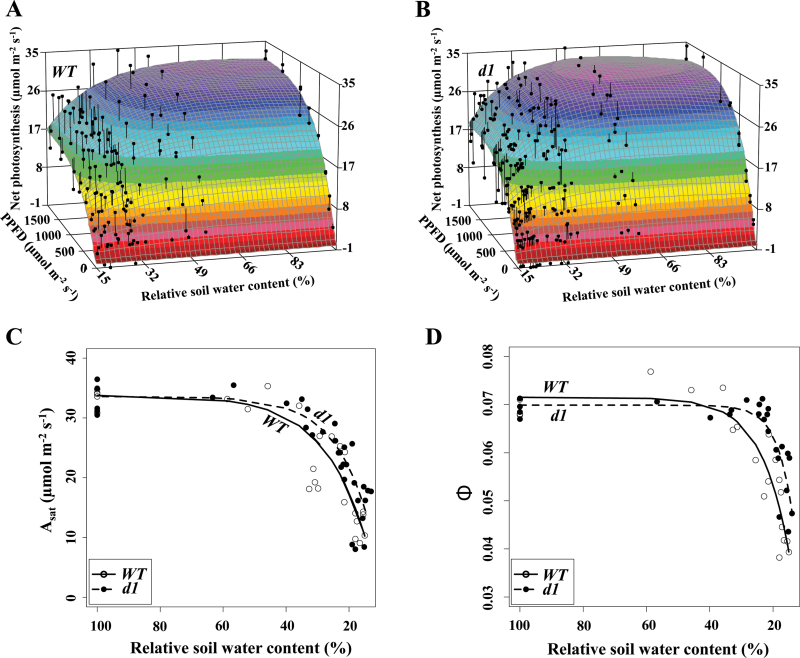
*A*/*I* curves in relation to relative soil water content reveal a greater effect of water limitation in the wild type (WT). (A and B) 3D response from fitting photosynthetic light response curves (*A*/*I*) as soil dries out reveals a greater effect of drought on the *A*/*I* responses of the WT, which can be dissected into parameters derived from the *A*/*I* relationship. Data were fitted using a 3D *ln* cumulative function (*r* ^2^_WT_=0.85, df_wt_=239; *r* ^2^_*d1*_=0.89, df_*d*1_=320). (C and D) Two parameters derived from the *A*/*I* curves. (C) The maximal photosynthesis at saturating light conditions (*A*_sat_) was more affected by water limitation in the WT than in *d1*. (D) The apparent quantum yield (Φ), which is effectively the slope of the *A*/*I* curve, indicates a steeper decline of photosynthesis in the WT than in *d1*. Data in both (C) and (D) were fitted to saturating exponential functions (Supplementary Tables S8, S9). (This figure is available in colour at *JXB* online.)

**Fig. 6.**
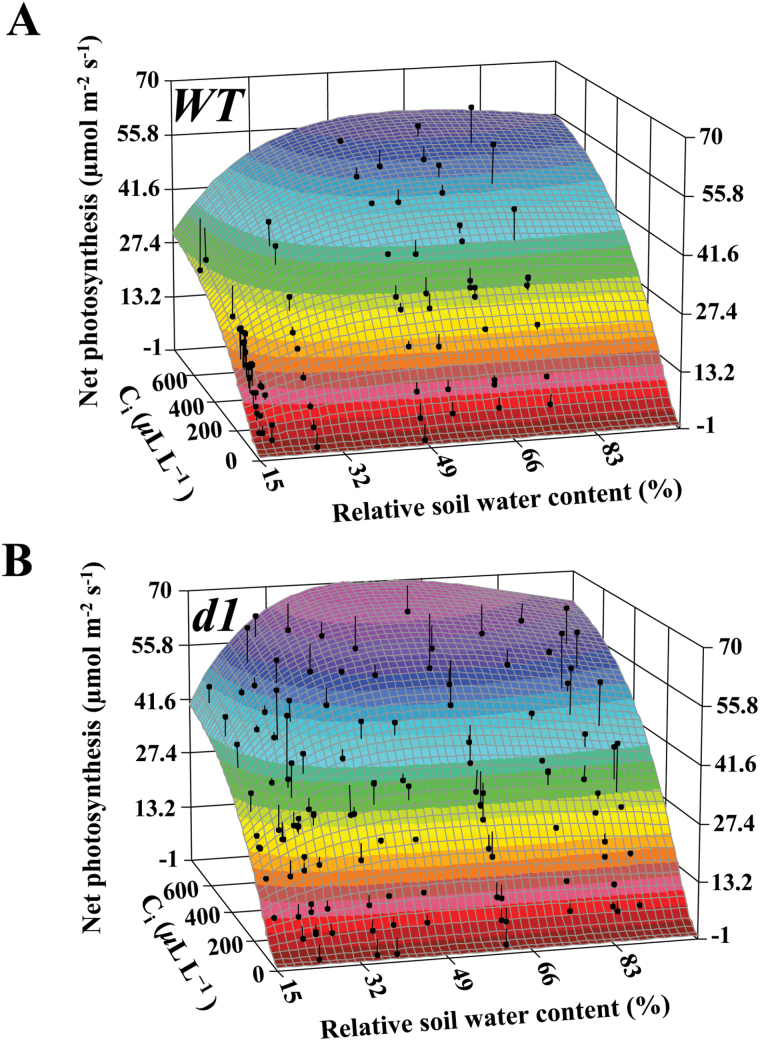
*A*/*C*_i_ curves in relation to relative soil water content (RSWC) reveal a greater non-stomatal limitation of photosynthesis in the wild type (WT) as the RSWC decreases from 30%. 3D fitting of *A*/*C*_i_ curves against RSWC for (A) the WT and (B) *d1* as soil dries out reveals a differential response of the two genotypes in photosynthetic capacity in response to water limitation. As the RSWC decreases from 100% to 50%, there is an increase in the maximal photosynthetic capacity of *d1*, which exhibits higher maximal photosynthetic capacity (*A*_max_) as RSWC approaches 50%. As drought becomes more severe (RSWC ≤30%), there is a decrease in photosynthetic capacity in *d1*, which results from non-stomatal limitation that is more severe in the WT than in *d1* (*r* ^2^_WT_=0.93, df_WT_=104; *r* ^2^_*d1*_=0.95, df_*d1*_=187). (This figure is available in colour at *JXB* online.)

We note that minimum soil water content is close to 10% for both genotypes (parameter b, Supplementary Table S1), and is first achieved 12 d after initiation of drought treatment for both genotypes. Given that *d1* but not WT plants then exhibit drought tolerance for an additional week (Fig. 1B, C), in order to avoid comparing with the WT the data from *d1* plants that have endured conditions close to minimum RSWC (10%) for an entire extra week, we focus on variables measured over the span of RSWC from soil field capacity (100% RSWC; day 0 for both genotypes) to 15% RSWC (day 11 for both genotypes). While in Figs 2–6 we compare genotypes as a function of RSWC, it should be kept in mind that the differences between genotypes in the measured parameters are of a greater magnitude when plotted against time (e.g. compare Fig. 1C versus D) than when such differences are depicted against RSWC.

Data for biomass and for leaf temperature exhibited an associated large variance, and so could not be fitted effectively using an appropriate model while attaining a biologically meaningful bias–variance trade-off. For that reason, these data were grouped into either absence of drought, for individuals for which RSWC was > 50%, or water limited, for those in which RSWC was between 50% and 15%; the data were then plotted as barplots constructed using the barplot function of Rgui (R Development Core Team, 2015). Statistical comparison between genotypes was conducted using the Student’s *t*-test. In the figures, asterisks denote *P<0.05, **P <0.01, and ***P<0.001.

## Results

### The *RGA1* mutant, *d1*, exhibits reduced sensitivity to drought stress

After plants were grown under well-watered conditions for 60 d, soil was allowed to dry progressively from what was defined as day 0 of drought treatment. For both genotypes, the decline of RSWC as a function of time exhibited a rapid initial decrease, with the soil losing ~25% of water content over the first day of treatment (Fig. 1A; first derivative maximum absolute value, Supplementary Table S1). Even during early vegetative growth, WT plants are bigger than *d1* plants (Fig. 1B). Accordingly, WT plants would be expected to take up water at a higher rate from the soil than *d1* plants, at least under well-watered conditions, but little difference was actually seen in soil drying for the two genotypes (Fig. 1A; Supplementary Table S1). This can be attributed to our deliberate choice of a large ratio of soil volume to biomass in these studies: the absence of any statistical difference between the curves in Fig. 1A demonstrates that transpirational withdrawal of water from the soil by the small plants of both genotypes is minor compared with soil evaporative water loss. Therefore, drought stress treatments were identical for the two genotypes.

Figure 1B illustrates the greater drought tolerance of a representative *d1* plant after progressive soil drying for 14 d. Leaves of the *d1* plant remain dark green and erect as compared with the leaf yellowing and turgor loss seen in the WT plant. Figure 1C displays the decline in net photosynthetic rates as drought progressed over time, starting from day 0, defined as the last day plants were watered to soil field capacity (60 d after emergence, 100% RSWC). The figure incorporates two different fittings as the data from day 0 of drought treatment (60 d after emergence) to day 4 were best fit to a linear model while the data from day 4 onward were best fit to a saturating exponential model (Supplementary Tables S2, S3). Between day 0 and day 4, net photosynthesis was constant for the WT, while photosynthesis slightly but significantly increased in *d1* as shown by the increased positive slope in the fit (parameter b, Supplementary Table S2; see the Discussion). From day 4 of drought onward, photosynthetic rates in both genotypes gradually decreased, but this response was significantly more severe in the WT (first derivative maximum absolute value, Supplementary Table S3) with the consequence that the net compensation point (at which net carbon fixation ceases) was reached 1 week earlier in WT (day 11) than in *d1* plants (day 18) as shown in the *x*-intercept parameter in the fit (parameter c, Supplementary Table S3). As is evident from Fig. 1C, at the point in time when the photograph of Fig. 1B was taken, 14 d after drought onset, the average WT plant had already ceased net carbon fixation several days previously, whereas the average *d1* plant would not reach the CO_2_ compensation point for several more days.

Figure 1D shows photosynthesis as a function of RSWC rather than as a function of time. At 100% RSWC, no statistical differences in net photosynthesis were found between the two genotypes (Fig. 1D; parameter a, Supplementary Table S4). As RSWC decreased below 50%, photosynthesis declined in both genotypes, but this effect was significantly more severe in the WT; at 50% RSWC, photosynthesis was higher in *d1* than in the WT and then it decreased at a somewhat slower rate (Supplementary Table S5).

WT plants before the onset of drought (100% to 50% RSWC) exhibited significantly greater above-ground (*P*=0.027) biomass, non-significantly greater below-ground biomass (*P*=0.124), and identical below-ground to above-ground (root to shoot) biomass ratios (*P*=0.965) to those of *d1* (Fig. 2). Due to additional carbon fixation during the drought treatment, there was an increase in both above- and below-ground biomass in both genotypes, while maintaining a difference in size between the WT and *d1* (Fig. 2A, B; *P*_above-ground_<0.001; *P*_below-ground_=0.005). During drought, both the WT and *d1* increased the proportion of growth allocated to the root system (Fig. 2B), resulting in an increase in the root to shoot ratio (Fig. 2C). While this ratio did not differ between genotypes in the absence of drought, droughted *d1* plants showed a significantly higher root to shoot ratio than the WT under the identical drought conditions (*P*<0.001, Fig. 2C).

### *d1* exhibits higher stomatal conductance and lower stomatal limitation of photosynthesis but the same transpirational water loss as the WT, which can be explained by a higher leaf temperature in the WT

Under well-watered conditions, stomatal conductance (*g*_s_) was higher in *d1* as compared with the WT (Fig. 3A; parameter a, Supplementary Table S6). Stomatal conductance decreased in both genotypes as a result of stomatal closure, and under severe drought both genotypes exhibited a similar *g*_s_ (Fig. 3A; parameter c, Supplementary Table S6). However, transpirational water loss was identical between the WT and *d1* under both well-watered and water-limited conditions (Fig. 3B; Supplementary Table S7).

The fact that *d1* had a higher stomatal conductance than the WT but the WT lost water at a rate per unit area that is slightly greater than that exhibited by *d1* can be explained by the fact that the WT had elevated leaf temperatures between 100% and 50% RSWC (*P*=0.005; Fig. 4A, B) and from 50% to 15% although non-significantly (*P*=0.079) so. As a result, the driving force for water loss, vapor pressure deficit (VPD), was greater for the WT than for *d1*. As soil dried out and stomata closed, leaf temperature increased in both genotypes due to stomatal closure (Fig. 4A, B), as a result of the reduction in transpirational cooling, but leaf temperature was always higher in the WT (Fig. 4B).

### Both stomatal and non-stomatal limitation of photosynthesis resulting from drought are less severe in *d1* plants

Supraoptimal leaf temperatures can have direct deleterious effects on photosynthesis (Berry and Bjorkman, 1980; Crafts-Brandner and Salvucci, 2000). Light response curves (*A*/*I*) were not different between the WT and *d1* under well-watered conditions (Fig. 5), but, as water stress became severe, *A*/*I* curves (Figs. 5A, B) revealed that maximal photosynthesis under light-saturating conditions (*A*_sat_; Fig. 5C) decreased at a faster rate in the WT than in *d1* (first derivative maximum absolute value, Supplementary Table S8) and apparent quantum yield (Φ; Fig. 5D) decreased earlier in the WT than in *d1* (parameter b, Supplementary Table S9). The decline in *A*_sat_ was first observed at ~50% RSWC for both genotypes, prior to the onset of non-stomatal limitation of photosynthesis at ~30% RSWC (see Fig. 6), suggesting that the decline was primarily the result of stomatal limitation of CO_2_ availability. In contrast, a decrease in quantum yield became evident only as RSWC decreased beyond 30% (Fig. 5D), suggesting that beyond 30% RSWC both stomatal and non-stomatal limitation of photosynthesis were occurring.

From initiation of the drought treatment until 40% RSWC, photosynthetic rates were in the range of 20–25 μmol m^−2^ s^−1^ for both genotypes (Fig. 1C, D; parameter a, Supplementary Tables S2, S4). Under saturating light conditions and ambient CO_2_, as shown in the *A*/*I* curves, photosynthesis (*A*_sat_) reached values of 34 μmol m^−2^ s^−1^ for both genotypes (Fig. 5C; parameter a, Supplementary Table S8). Next, to determine *A*_max_, *A*/*C*_i_ responses were obtained at saturating light intensities to avoid light limitation of the maximum rate of Rubisco carboxylation under saturating intercellular CO_2_ concentrations. By plotting a 3D model of assimilation at saturating light intensities (1500 μmol m^−2^ s^−1^), as a function of intercellular CO_2_ concentration in relation to RSWC (*A*/*C*_i_/RSWC curves; Fig. 6), it is apparent that at 100% RSWC, photosynthesis under saturating intercellular CO_2_ (intercellular CO_2_ partial pressure of 800 μl l^−1^) conditions reached values close to 55 μmol m^−2^ s^−1^ for both genotypes (Fig. 6). The values of *A* that we report in Fig. 6 for well-watered conditions are similar to those described previously in the literature for an *A*/C_i_ curve in well-watered rice under saturating light intensities (Takai *et al.*, 2013). As soil dried to values close to 50% RSWC, the WT exhibited a gradual decline in *A*_max_, while *d1* actually exhibited an increase in maximum assimilation (light purple region in Fig. 6B) prior to a sharp decrease as drought severity increased. This is consistent with the pattern observed for carbon fixation under ambient growing conditions (Fig. 1C, D) and *A*/*I* curves (Fig. 5).

## Discussion

### The *RGA1* mutant, *d1*, exhibits sustained photosynthesis and increased resistance to drought stress

Rice is grown either in irrigated (lowland) or in rainfed conditions (upland or lowland). Rice is particularly sensitive to water shortage (Tanguilig *et al.*, 1987; Miyamoto *et al.*, 2001), and water limitation is the biggest constraint to yield production in rainfed rice (Tuong and Bouman, 2003). In any cultivation system, water supply is of major importance; in the case of irrigated systems because of its elevated usage and cost, and in rainfed conditions because of the dependence of plants on a water supply that is unpredictable and often discontinuous. Accordingly, breeding for rice varieties for drought tolerance is becoming an increasingly important target (Fukai and Cooper, 1995; Passioura, 2012).

It is evident from visual observation that *d1* plants endure longer through drought conditions in comparison with the WT (Fig. 1B). Increased relative allocation of biomass to roots in *d1* (Fig. 2) may have contributed (Weiner and Solbrig, 1984) to the ability to sustain photosynthesis under water limitation (Fig. 1C). It is interesting to note that, regardless of whether the photosynthetic rate is plotted relative to time after drought initiation (Fig. 1C) or relative to RSWC (Fig. 1D), there was an increase in photosynthesis in *d1* as soil dried from saturating conditions to ~50% RSWC, which occurs at 4 d after initiation of drought (see also Figs 5 and 6). The basis for this increase is unknown, but might be related to decreased tolerance by *d1* of hypoxic soil conditions associated with high RSWC. Modern rice varieties are sensitive to anaerobic conditions in the soil caused by flooding during germination and early vegetative growth (Miro and Ismail, 2013) and RGA1 has been previously suggested to regulate ethylene-mediated hypoxia signaling in rice (Steffens and Sauter, 2010). The longer survival of *d1* plants may be of particular agronomic importance at the young vegetative stage, as assayed here. In rice, drought during the seedling stage affects survival and leaf and shoot growth, modifying light interception, resource uptake, and finally reproductive yield following recovery (Boonjung and Fukai, 1996).

Since very little is known concerning how disruption of heterotrimeric G protein signaling affects the physiology of any crop species, after documenting the greater drought tolerance of the *d1* mutant, our further objective was to dissect the physiological causes and processes that resulted in this tolerance.

### The effects of stomatal limitation on photosynthesis are less severe in *d1* plants

Stomatal conductance was statistically greater in *d1* than in the WT until ~25% RSWC, resulting in lower stomatal limitation of photosynthesis in this G protein mutant over much of the drought treatment, particularly between 50% and 25% RSWC. Notably, this increased stomatal conductance (Fig. 3A) did not come at the cost of greater transpirational water loss (Fig. 3B). Our data (Fig. 4) suggest that these initially puzzling results can be reconciled by the fact that increased leaf temperatures prevailed in the WT in comparison with *d1*, which resulted in a greater driving force (VPD) for transpirational water loss in the WT (Supplementary Fig. S1; Supplementary Tables S10, S11). Transpiration is a function of both stomatal conductance and VPD. Due to the greater stomatal conductances yet lower leaf temperatures in *d1* than in the WT, transpiration was equal in the two genotypes, but stomatal limitation of CO_2_ uptake was lower in *d1*.

Given equal transpiration, and thus equal transpirational cooling in the two genotypes, the elevated leaf temperatures in the WT must have originated from another source, which we hypothesize to be greater interception of near infrared radiation by the WT than by *d1*. This hypothesis is consistent with the less erect orientation of leaves and tillers in the WT as compared with *d1*. It is of interest that, in upland rice, Hirayama *et al.* (2006) observed that leaf temperature was negatively correlated with yield under water-limited conditions, and so these authors proposed leaf temperature as a convenient indicator for cultivar selection in upland rice breeding (Garrity and O’Toole, 1995; Hirayama *et al.*, 2006).

### The effects of non-stomatal limitation of photosynthesis caused by drought are less severe in *d1* plants

Drought can limit photosynthesis by both stomatal limitation and non-stomatal mechanisms. Stomatal limitation arising from stomatal closure is the main limitation to C_3_ photosynthesis at moderate drought stress (Flexas and Medrano, 2002). As drought becomes more severe, non-stomatal factors become the main limitation on photosynthetic CO_2_ assimilation (Flexas and Medrano, 2002). Consistent with this paradigm, in our study, stomatal limitation of photosynthesis occurred first. Stomatal limitation was associated with a decrease in maximum light-saturated photosynthesis, which initiated at a higher RSWC in the WT than in *d1*. The decrease of apparent quantum yield in response to soil desiccation also initiated at a higher RSWC in the WT than in *d1*, and the decrease was more abrupt (Fig. 5D), consistent with the greater susceptibility of the WT to drought.

As drought became more severe, non-stomatal limitation of photosynthesis also occurred. Non-stomatal limitation of photosynthesis was confirmed in *A*/*C*_i_ curves (Fig. 6), which revealed that when RSWC was 100%, both genotypes exhibited similar photosynthetic capacities. As RSWC decreased, there was an initial increase in photosynthesis at the saturating intercellular CO_2_ concentration in *d1*, suggestive of an increase in maximal photosynthetic capacity, consistent with the increase in assimilation seen under ambient conditions in Fig. 1C and D. As RSWC decreased from 50%, maximal photosynthetic capacity (*A*_max_; Fig. 6) decreased in both genotypes, but more rapidly for the WT, such that as RSWC reached 15%, approaching minimum soil water-holding capacity (10%), the maximal photosynthetic capacity of *d1* plants (41.6 μmol m^−2^ s^−1^) was considerably higher than that of WT plants (27.4 μmol m^−2^ s^−1^). This indicates that non-stomatal limitation of photosynthesis occurred earlier and at higher RSWC in the WT than in *d1*. WT plants may have experienced more severe non-stomatal limitation of photosynthesis at least in part due to their higher leaf temperatures: drought and high temperature have a synergistic negative effect on photosynthesis in many plant species (Shah and Paulsen, 2003; Mittler, 2006). High temperatures can reduce net photosynthesis by increasing photorespiration and by inhibiting repair of photosystem II damage resulting from photoinhibition (Berry and Bjorkman, 1980; Ögren and Öquist, 1985; Takahashi and Murata, 2008; Takahashi and Badger, 2011).

It is useful to recall that water loss from the soil was very rapid until ~30% RSWC and then the rate of water loss moderated (Fig. 1A). Scaling this into time after the drought treatment commenced, the point at which non-stomatal limitation of photosynthesis started to occur was about day 7 in the WT. Severe effects of drought were evident in the WT soon thereafter, as net carbon assimilation approached zero at about day 11 (Figs 1C, 3A). In contrast, the same effects occurred many days later in *d1*, with loss of net carbon assimilation not reached until about day 18 in this genotype (Fig. 1C).

### Differential water use efficiency does not reflect drought susceptibility

Up to this point, we have discussed carbon and water relationships based on a per unit leaf area basis. From a whole-plant perspective, it is evident, given the dwarf stature of *d1* (Fig. 2A), that the WT fixes more carbon and loses more water than *d1*, and that these genotypic differences are large. In interspecific competition, species that use more of a given resource such as water will outcompete other species, as the former will have an increased relative growth rate. However, in the case of monocultures such as prevail in the cultivation of most crops, including rice, ideal conditions involve a decreased asymmetry in resource utilization between individuals of the population (Weiner and Solbrig, 1984). Accordingly, useful rice cultivars resistant to drought would not be those that successfully compete for the most water, such as the WT, but rather those that need the least water and make equal or more efficient use of it, such as *d1*.

In summary, we have shown that mutation of the Gα subunit of the rice heterotrimeric G protein results in significantly improved drought tolerance. This improved drought tolerance is manifested in the prolonged survival under drought of *d1* plants as compared with WT plants, and is associated with higher stomatal conductance, lower leaf temperatures, and delayed onset of both stomatal and non-stomatal limitation of photosynthesis in *d1* as compared with the WT. In the future it will be of interest to unravel the exact molecular mechanisms by which the *d1* mutation modifies cellular signaling in rice to produce the physiological alterations documented here.

Drought is an important limiting parameter for seedling growth and canopy establishment in rainfed rice (O’Toole *et al.*, 1978), and even a few days of water limitation can severely affect seedling survival, growth, and subsequent yield (Boonjung and Fukai, 1996). Accordingly, plants harboring this natural *d1* mutation may hold promise for breeding programs targeted at improved seedling establishment and survival. Despite biotechnological advances, natural crop mutants, as is the case for *d1*, are still especially valued because they maintain substantially higher consumer acceptance. In addition, the improved drought tolerance of *d1* plants suggests that a broader emphasis on modulating heterotrimeric G protein signaling in rice could be useful in breeding and biotechnological manipulations focused on improving stress tolerance in this essential crop species.

## Supplementary data

Supplementary data are available at *JXB* online.

Data S1.

**Figure S1.** Vapor pressure deficit (VPD) increases as relative soil water content (RSWC) in the soil decreases, and the WT exhibits a higher VPD than *d1* for all RSWC values.

**Table S1.** Model fit and parameter estimate for the decay model representing the relationship between relative soil water content and time after drought treatment started (Fig. 1A).

**Table S2.** Model fit and parameter estimate for the linear model representing the relationship between photosynthesis and time after watering was withheld until day 4 (Fig. 1C).

**Table S3.** Model fit and parameter estimate for the saturating exponential model representing the relationship between photosynthesis and time from day 4 onward after watering was withheld (Fig. 1C).

**Table S4.** Model fit and parameter estimate for the linear relationship between photosynthesis and relative water content after water was withheld until 50% relative soil water content (Fig. 1D).

**Table S5.** Model fit and parameter estimate for the saturating exponential model representing the relationship between photosynthesis and relative soil water content from 50% to 15% relative soil water content (Fig. 1D).

**Table S6.** Model fit and parameter estimate for the saturating exponential model representing the relationship between stomatal conductance and relative soil water content (Fig. 3A).

**Table S7.** Model fit and parameter estimate for the saturating exponential model representing the relationship between transpiration and relative soil water content (Fig. 3B).

**Table S8.** Model fit and parameter estimate for the saturating exponential model representing the relationship between *A*_sat_ and relative soil water content (Fig. 5C).

**Table S9.** Model fit and parameter estimate for the saturating exponential model representing the relationship between apparent quantum yield and relative soil water content (Fig. 5D).

**Table S10.** Model fit and parameter estimate for the linear relationship between vapor pressure deficit (VPD) and relative soil water content after water was withheld until 25% relative soil water content (Supplementary Fig. S1).

**Table S11.** Model fit and parameter estimate for the linear relationship between vapor pressure deficit (VPD) and relative soil water content from 25% to 15% after watering was withheld (Supplementary Fig. S1).

Supplementary Data
