# Supplementary material for: The α-subunit of the rice heterotrimeric G protein, RGA1, regulates drought tolerance during the vegetative phase in the dwarf rice mutant d1
Source: J Exp Bot. 2016 May 18;67(11):3433–43. doi: 10.1093/jxb/erw183 (PMC4892740; doi:10.1093/jxb/erw183)
Supplement: Supplementary Data [file supp_67_11_3433__index.html]

The α-subunit of the rice heterotrimeric G protein, RGA1, regulates drought tolerance during the vegetative phase in the dwarf rice mutant d1 — The α-subunit of the rice heterotrimeric G protein, RGA1, regulates drought tolerance during the vegetative phase in the dwarf rice mutant d1 — Supplementary Data 

# The α-subunit of the rice heterotrimeric G protein, RGA1, regulates drought tolerance during the vegetative phase in the dwarf rice mutant *d1*

## Supplementary Data

Data files

- Supplementary\_data\_S1\_Supplementary\_Figure\_S1\_Tables\_S1\_S11.pdf - Supplementary Data
