# Supplementary material for: The α-subunit of the rice heterotrimeric G protein, RGA1, regulates drought tolerance during the vegetative phase in the dwarf rice mutant d1
Source: J Exp Bot. 2016 May 18;67(11):3433–43. doi: 10.1093/jxb/erw183 (PMC4892740; doi:10.1093/jxb/erw183)
Supplement: Supplementary Data [file supp_erw183_Supplementary_data_S1_Supplementary_Figure_S1_Tables_S1_S11.pdf]

The α-subunit of the rice heterotrimeric G-protein, RGA1, regulates drought tolerance during the vegetative phase in the dwarf rice mutant *d1*

Ángel Ferrero-Serrano[1] and Sarah M. Assmann[1]

SUPPLEMENTARY DATA S1

Data fitting

All data were fitted to one of three functions depending on the data distribution: a saturating exponential function, a decay function, or a linear function. Outliers were removed after inspection of the standardized residuals of the individual data points for each fit if the Y value was considered unusual given its explanatory variables and thus attributable to technical or experimenter error. In the worst case-scenario, removed outliers constituted less than 5.7% of the data for WT and 4.6% for *d1*. For simplicity, we used the same function for the same type of distribution, even in cases a where a more complicated (but less physiologically explicable) distribution would improve the fit. We concluded from examination of the lack of fit values (see Supplementary Tables) generated from use of the above three distributions that these distributions represent a good fit for the data. (In general lack of fit values less than 0.5 indicate poor fits).

The resultant fitted equation for the saturating exponential function was:

$$y = a(1 - e^{-b(x-c)})$$

where $\boldsymbol{x}$ is the explanatory variable and $\boldsymbol{y}$ is the dependent variable, $\boldsymbol{a}$ represents the upper asymptote, **b** is a shape parameter and **c** represents the x-intercept. The period of maximal rate of decrease in y was calculated from the absolute value of the 1st derivative maximum of the fitted function.

The resultant fitted equation for the decay function was:

$$y = b + (a - b)e^{-cx}$$

where $\boldsymbol{x}$ is the explanatory variable and $\boldsymbol{y}$ is the dependent variable, $\boldsymbol{a}$ represents the lower asymptote, $\boldsymbol{b}$ is a shape parameter and $\boldsymbol{c}$ represents the $\boldsymbol{y}$-intercept. The period of maximal rate of increase in **y** was calculated from the absolute value of the 1st derivative minimum of the fitted function.

For the fitted linear equation:

$$y = bx + a$$

where $\boldsymbol{x}$ is the explanatory variable and $\boldsymbol{y}$ is the dependent variable. The slope of the line is $\boldsymbol{b}$, and $\boldsymbol{a}$ is the **y** intercept.

SUPPLEMENTARY FIGURE

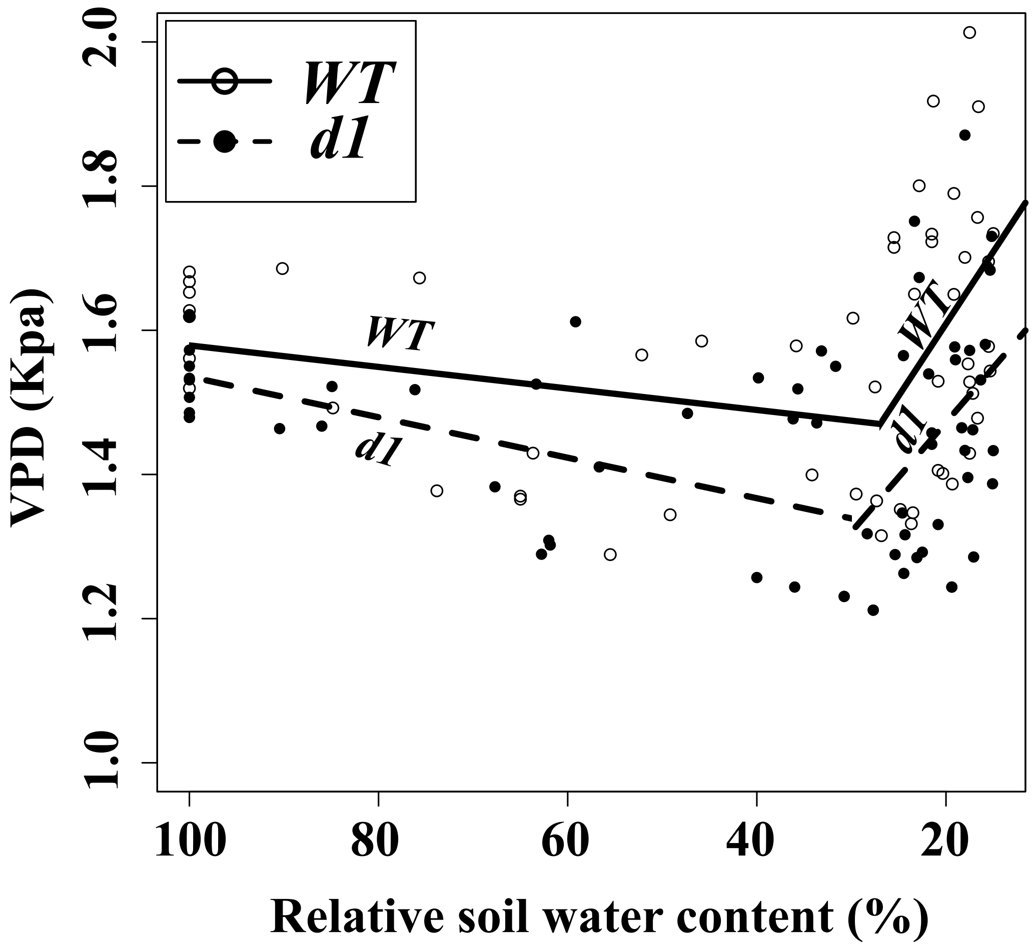

Figure S1: Vapour pressure deficit (VPD) increases as relative soil water content (RSWC) in the soil decreases and WT exhibits a higher VPD than *d1* for all RSWC values. Fits were made using linear fits for the response until 25% RSWC (Supplementary Table S10) and then from 25% to 15% (Supplementary Table S11).

SUPPLEMENTARY TABLES

Table S1. Model fit and parameter estimate for the decay model representing the relationship between relative soil water content and time after drought treatment started (Fig. 1A).

Genotype	a	b	c	df	r^2	1st derivative maximum absolute value
**WT**	100.33 (±0.82)	9.64 (±1.05)	0.28 (±0.01)	68	0.99	25.66
d1	100.09 (±0.74)	9.57 (±0.76)	0.24 (±0.01)	75	0.99	22.30

Table S2. Model fit and parameter estimate for the linear model representing the relationship between photosynthesis and time after watering was withheld until day 4 (Fig. 1C).

Genotype	a	b	df	r^2
**WT**	20.18 (±0.34)	0.02 (±0.16)	26	0.006
d1	20.18 (±0.35)	0.57 (±0.16)	20	0.310

Table S3. Model fit and parameter estimate for the saturating exponential model representing the relationship between photosynthesis and time from day 4 onward after watering was withheld (Fig. 1C).

Genotype	a	b	c	df	r^2	1st derivative maximum absolute value
**WT**	34.95 (±13.43)	-0.12 (±0.07)	10.68 (±0.22)	37	0.91	4.63
d1	29.15 (±3.43)	-0.11 (±0.02)	17.63 (±0.39)	67	0.87	2.94

Table S4. Model fit and parameter estimate for the linear relationship between photosynthesis and relative soil water content after water was withheld until 50% relative soil water content (Fig. 1D).

Genotype	a	b	df	r^2
**WT**	21.17 (±1.15)	-0.01 (±0.013)	21	0.01
d1	25.05 (±1.28)	0.01 (±0.017)	25	0.32

Table S5. Model fit and parameter estimate for the saturating exponential model representing the relationship between photosynthesis and relative soil water content from 50% to 15% relative soil water content (Fig. 1D).

Genotype	a	b	c	df	r^2	1st derivative maximum absolute value
**WT**	21.99 (±3.712)	0.09 (±0.06)	9.20 (±4.45)	31	0.409	1.10
d1	23.25 (±1.062)	0.10 (±0.03)	7.07 (±2.569)	38	0.714	1.06

Table S6. Model fit and parameter estimate for the saturating exponential model representing the relationship between stomatal conductance and relative soil water content (Fig. 3A).

Genotype	a	b	c	df	r^2	1st derivative maximum absolute value
**WT**	0.27 (±0.013)	0.12 (±0.03)	12.92 (±1.56)	56	0.54	0.02
d1	0.31 (±0.012)	0.10 (±0.02)	11.4 (±1.53)	65	0.65	0.02

Table S7. Model fit and parameter estimate for the saturating exponential model representing the relationship between transpiration and relative soil water content (Fig. 3B).

Genotype	a	b	c	df	r^2	1st derivative maximum absolute value
**WT**	4.59 (±0.15)	0.09 (±0.19)	11.13 (±1.00)	52	0.71	0.28
d1	4.49 (±0.14)	0.09 (±0.02)	10.64 (±1.19)	65	0.72	0.27

Table S8. Model fit and parameter estimate for the saturating exponential model representing the relationship between between A_{sat} and relative soil water content (Fig. 5C).

Genotype	a	b	c	df	r^2	1st derivative maximum absolute value
**WT**	34.04 (±1.88)	0.06 (±0.01)	8.24 (±2.60)	22	0.81	1.32
d1	34.02 (±1.61)	0.07 (±0.01)	7.38 (±2.21)	30	0.76	1.67

Table S9. Model fit and parameter estimate for the saturating exponential model representing the relationship between apparent quantum yield and relative soil water content (Fig. 5D).

Genotype	a	b	c	df	r^2	1st derivative maximum absolute value
**WT**	0.07 (±0.002)	0.11 (±0.03)	7.92 (±2.40)	17	0.84	0.004
d1	0.07 (±0.002)	0.20 (±0.06)	8.34 (±2.10)	22	0.71	0.004

Table S10. Model fit and parameter estimate for the linear relationship between vapour pressure deficit (VPD) and relative soil water content after water was withheld until 25% relative soil water content (Supplementary Fig. S1).

Genotype	a	b	df	r^2
**WT**	1.43 (±0.06)	0.001 (±0.001)	25	0.06
d1	1.25 (±0.04)	0.003 (±0.001)	36	0.35

Table S11. Model fit and parameter estimate for the linear relationship between vapour pressure deficit (VPD) and relative soil water content time from 25% to 15% after watering was withheld (Supplementary Fig. S1).

Genotype	a	b	df	r^2
**WT**	2.09 (±0.26)	-0.019 (±0.013)	26	0.07
d1	1.78 (±0.19)	-0.015 (±0.009)	28	0.05
